# Rapid Identification of ASFV, CSFV and FMDV from Mongolian Outbreaks with MinION Short Amplicon Sequencing

**DOI:** 10.3390/pathogens12040533

**Published:** 2023-03-29

**Authors:** Dashzeveg Bold, Jayme A. Souza-Neto, Delgerzul Gombo-Ochir, Natasha N. Gaudreault, David A. Meekins, Chester D. McDowell, Batsukh Zayat, Juergen A. Richt

**Affiliations:** 1 Department of Diagnostic Medicine/Pathobiology, College of Veterinary Medicine, Kansas State University, Manhattan, KS 66506, USA; 2 State Central Veterinary Laboratory, Ulaanbaatar 17029, Mongolia; 3 Institute of Veterinary Medicine, Mongolian University of Life Sciences, Ulaanbaatar 17029, Mongolia

**Keywords:** transboundary animal diseases, African swine fever virus, classical swine fever virus, foot-and-mouth disease virus, point-of-care diagnostics, MinION, short amplicon sequencing

## Abstract

African swine fever virus (ASFV), classical swine fever virus (CSFV), and foot-and-mouth disease virus (FMDV) cause important transboundary animal diseases (TADs) that have a significant economic impact. The rapid and unequivocal identification of these pathogens and distinction from other animal diseases based on clinical symptoms in the field is difficult. Nevertheless, early pathogen detection is critical in limiting their spread and impact as is the availability of a reliable, rapid, and cost-effective diagnostic test. The purpose of this study was to evaluate the feasibility to identify ASFV, CSFV, and FMDV in field samples using next generation sequencing of short PCR products as a point-of-care diagnostic. We isolated nucleic acids from tissue samples of animals in Mongolia that were infected with ASFV (2019), CSFV (2015), or FMDV (2018), and performed conventional (RT-) PCR using primers recommended by the Terrestrial Animal Health Code of the World Organization for Animal Health (WOAH). The (RT-) PCR products were then sequenced in Mongolia using the MinION nanopore portable sequencer. The resulting sequencing reads successfully identified the respective pathogens that exhibited 91–100% nucleic acid similarity to the reference strains. Phylogenetic analyses suggest that the Mongolian virus isolates are closely related to other isolates circulating in the same geographic region. Based on our results, sequencing short fragments derived by conventional (RT-) PCR is a reliable approach for rapid point-of-care diagnostics for ASFV, CSFV, and FMDV even in low-resource countries.

## 1. Introduction

Animals provide food and other essential resources to most of the world’s inhabitants. Therefore, the World Organization for Animal Health (WOAH) and the Food and Agriculture Organization of the United Nations (FAO) have cooperated in the Global Framework for the Progressive Control of Transboundary Animal Diseases (GF-TADs) to mitigate the threat of such diseases to the global food supply, safe trade, and livelihoods [1]. TADs are highly infectious and transmissible animal diseases that have the potential to cross borders and bring significantly negative socioeconomic and public health-associated consequences to the affected areas. In this context, identification of the factors contributing to disease pathogenesis and spread, with improved diagnostics and cost-effectiveness of preventive measures is crucial [2]. Outbreaks of several TADs including foot-and-mouth disease (FMD) [3], classical swine fever (CSF), and African swine fever (ASF), which resulted in significant economic losses, were recently reported in Mongolia [4,5,6].

ASF is a highly fatal swine disease and no efficacious and safe vaccine is available. The causative agent of the disease, African swine fever virus (ASFV), is the sole member of the *Asfarviridae* family, genus *Asfivirus*. This enveloped virus has a large double-stranded DNA genome ranging in size from 170 to 190 kilobase pairs (kbp). ASFV is classified into 24 genotypes according to the sequence diversity of its major capsid protein p72, and into eight serotypes based on its CD2-like and C-type lectin proteins [7]. A highly virulent genotype II ASFV has recently emerged in many parts of Europe, Asia, and the Caribbean [8,9,10] since its introduction into Georgia in 2007 [4,11,12,13,14,15,16].

CSF is one of most critical viral TADs affecting swine, caused by the classical swine fever virus (CSFV). CSFV is an enveloped, positive sense, single-stranded RNA virus with a 12.5 kb genome; it is a member of the *Pestivirus* genus in the *Flaviviridae* family [17]. CSF outbreaks have been reported around the world and recently in Colombia, Brazil, Russia, South Korea, and Japan [18].

FMD [3] is a highly contagious viral disease affecting cloven-hoofed animals, caused by the foot-and-mouth disease virus (FMDV). FMDV is a non-enveloped virus with a positive-sense RNA genome that is a member of the *Aphthovirus* genus in the *Picornaviridae* family. FMDV comes in seven genetically distinct serotypes (O, A, C, SAT-1, SAT-2, SAT-3, and Asia-1) and multiple topotypes. Despite its discovery in 1897 as the first vertebrate-infecting virus [19] and the availability of vaccines, FMDV outbreaks are still occurring worldwide. The virus is estimated to circulate in approximately three quarters of the global livestock population, mainly in Africa, the Middle East, and Asia, as well as in a limited area of South America [1]. Approximately 2.5 billion doses of FMD vaccine are globally deployed each year in endemic countries, primarily in China and South America [20].

Standard TAD diagnostics are regulated by WOAH and multiple specific assays may be available for a single disease depending on its purpose [21]. Currently, conventional PCR and real-time quantitative PCR (qPCR) are recommended for ASFV molecular detection, while serological tests such as ELISA, indirect immunoperoxidase test (IPT), and immunoblotting test (IBT) are routinely used for ASF diagnosis [22]. The diagnosis of FMD is mainly performed by conventional RT-PCR and RT-qPCR, and both methods are officially recommended by WOAH. ELISA-based antigen and antibody detection tests are used to survey exposure to the virus or recovery from viral infection, respectively. For CSF, conventional RT-PCR or ELISA-based antigen and antibody detection tests are recommended for diagnosis [22], even though a number of validated RT-qPCR-based diagnostics are available [12,23,24,25].

In most cases, TAD outbreaks occur in rural areas of the respective countries, and samples from suspected animals are collected by inspectors of local veterinary laboratories. Testing is accomplished using basic diagnostic assays including (RT-) PCR, ELISA, and point-of-care (POC) tests [26]. Accurate and rapid diagnosis of TADs is critical during suspected outbreaks to allow for a fast and effective response, which can mitigate pathogen spread [27]. Rapid diagnosis may be hindered in many cases by poor reliability of some assays or the lack of laboratory infrastructure in rural veterinary laboratories, which ultimately results in significant delays in pathogen identification [28]. Therefore, the development of fast, sensitive, specific, and reliable POC diagnostics is pivotal for early detection and mitigation of TAD outbreaks by the appropriate regulatory authorities.

Here, we used the MinION portable sequencer to identify ASFV, CSFV, and FMDV isolates collected during previous outbreaks in Mongolia. Our data show that all three pathogens can be quickly and reliably identified via amplicon sequencing with WOAH validated methods. This methodology is capable of providing additional important information such as genotype/serotype/strain differentiation in a relatively short turnaround time that other molecular or serological diagnostic tests may not offer.

## 2. Materials and Methods

### 2.1. Samples and Extraction of Nucleic Acids

The ASFV [4], CSFV [5], and FMDV [3] samples were previously collected and identified at the State Central Veterinary Laboratory (SCVL) in Ulaanbaatar, Mongolia using OIE/WOAH-recommended methods; the results were confirmed by OIE/WOAH reference laboratories in the UK and Japan (Table 1).

### 2.2. Polymerase Chain Reaction (PCR) and Reverse-Transcription (RT-) PCR

**Figure 1:**
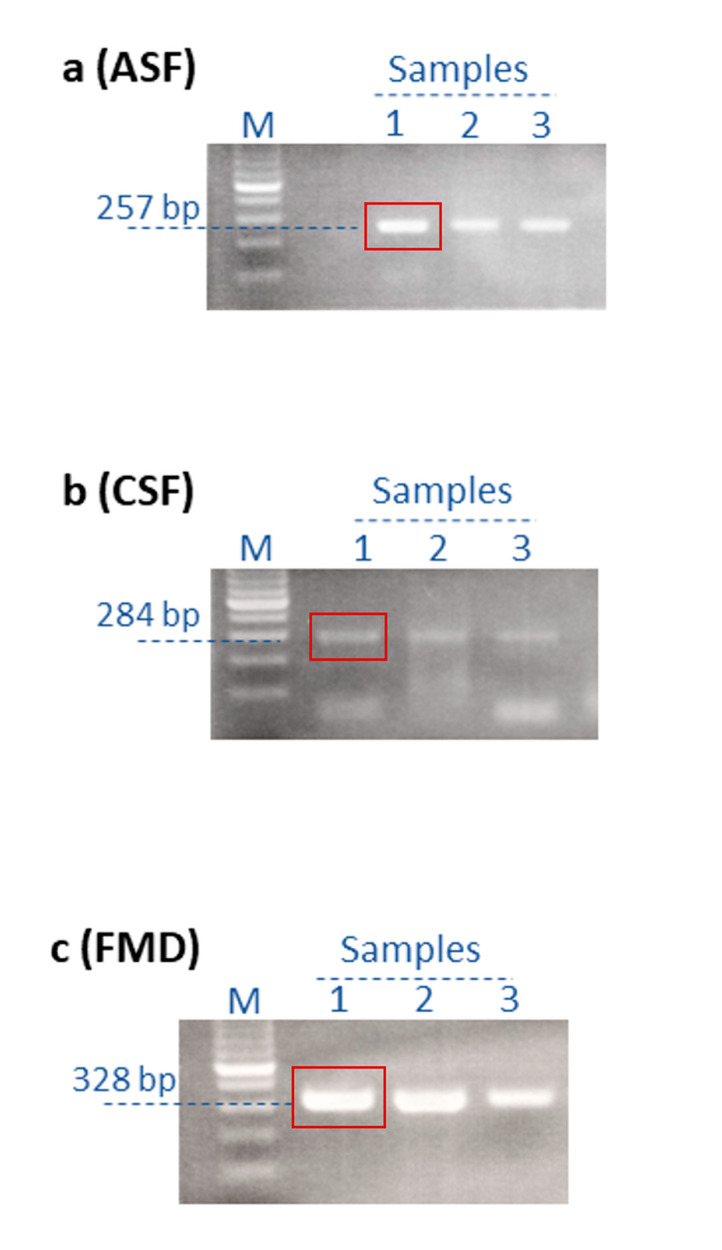
Agarose gel electrophoresis (2% agarose) of (RT)-PCR amplified products using virus-specific primer sets. The amplicons used for the MinION sequencing are in the respective red squares for ASF (**a**), CSF (**b**), and FMD (**c**). Lane M, 100 bp DNA size marker.

PCR (ASFV) and RT-PCR (CSFV and FMDV) assays were performed at the SCVL in Mongolia, following the Terrestrial Animal Health Code [22] of the World Organization for Animal Health (WOAH; founded as OIE). Three primer sets (Table 2) were used to amplify fragments of the ASFV gene encoding VP72, the CSFV IRES, or the FMDV 5′ UTR, respectively, and the amplicons were analyzed using gel electrophoresis (Figure 1) [22]. ASFV PCR reactions contained sample DNA (ASF samples) and DreamTaq Green PCR Master Mix (ThermoFisher, #K1082, CA, USA), whereas RT-PCR reactions contained sample RNA (either CSF or FMD samples) and SuperScript™ III One-Step RT-PCR System with Platinum™ Taq High Fidelity DNA Polymerase (Invitrogen, #12574035, CA, USA). The reactions were performed according to previously standardized conditions [22]. Amplicons were purified with AMPure XP beads (Beckman Coulter, #A63881, IN, USA) according to the manufacturer’s instructions prior to MinION library preparation.

### 2.3. Oxford Nanopore MinION Sequencing

Individual single-plex sequencing libraries were prepared with the Oxford Nanopore ligation sequencing kit (SQK-LSK109) for each virus according to the manufacturer’s instructions. Briefly, 200 µL of bead-purified amplicons were added to 16 µL of library mix and incubated at 20 °C for 5 min and 65 °C for 5 min. Sequencing adapter ligation was prepared with 60 µL of library mix, 25 µL of Ligation Buffer 25, 10 µL of NEBNext Quick T4 DNA Ligase, and 5 µL of Adapter Mix with a final volume of 100 µL. The sample was gently mixed by flicking the tube, spun down, and then incubated for 10 min at room temperature. Next, the MinION flow cell (model R9.4.1) was primed with the MinION Priming Kit prior to loading the libraries following the manufacturer’s instructions. Afterwards, 75 µL of a final mix (consisting of 37.5 µL of sequencing buffer (SQB), 25.5 µL of loading beads (LB), and 12 µL of amplicon library) was loaded dropwise onto the flow cell via the SpotON sample port for sequencing. Sequencing runs were controlled with the MinKNOW software following the instructions provided in the MinKNOW protocol. ASFV and CSFV amplicon libraries were sequenced individually using the same flow cell that was thoroughly washed between samples. About 10% (17,395) of the reads that were generated during CSFV library sequencing were assigned to ASFV likely due to carryover from one run to another, and, therefore, were disregarded from the analysis. The FMDV amplicon library was sequenced in a separate flow cell.

### 2.4. Sequencing Data Processing and Analysis

Sequencing reads were parsed into multiple FASTQ files with the MinKNOW control software. The data were initially analyzed using the cloud-based service EPI2ME (Metrichor). This analysis was performed using FASTQ WIMP (What’s in My Pot) workflow, which relies on a method called Centrifuge, a microbial classification engine (EPI2ME workflow) [29]. The sequencing data was also analyzed with CLC Genomic Workbench 20.0 (Qiagen, Carlsbad, CA, USA). For this, MinION sequencing reads were filtered to remove reads with a q-score < 7 and lengths < 200 bp and >400 bp. FASTQs were imported into the CLC software and parsed into individual sample files. The filtered reads were mapped to the respective reference genomes (ASFV: FR682468.2, CSFV: KY290453, FMDV: LC438823) using the CLC Genomics Workbench Map to Reference Module with a 50% length and 80% similarity mapping parameters. The obtained consensus sequences were extracted and compared to the NCBI nucleotide database using the *blastn* tool within the CLC Genomic Workbench for taxonomic identification and the top 100 hits were downloaded for phylogenetic analysis.

### 2.5. Sequence Alignment and Phylogenetic Analysis

The consensuses sequences were aligned with other sequences of the respective pathogen (ASFV, CSFV, or FMDV) using ClustalW and phylogenetic analyses were performed with MEGA 11 (Molecular Evolutionary Genetic Analysis) [30,31,32,33]. Phylogenetic trees were reconstructed with maximum likelihood and 1000 bootstrap replications.

## 3. Results

**Figure 2:**
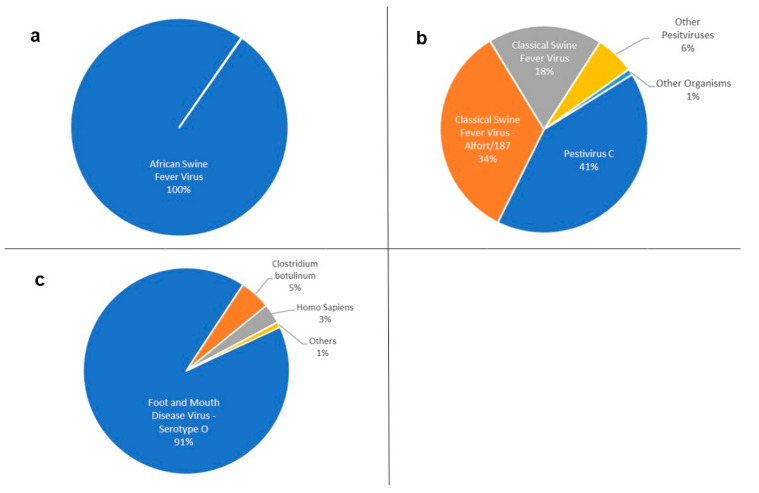
Relative distribution of reads in sequence data generated by nanopore sequencing of DNA from (RT-) PCR products of three pathogens, ASFV (**a**), CSFV (**b**), and FMDV (**c**). The pie-charts were created using Microsoft Office Excel 2021.

Using WOAH-recommended primer sets [22], we successfully amplified genomic fragments from ASFV *p72*, CSFV *IRES*, and FMDV 5′UTR, and further generated Oxford Nanopore MinION amplicon libraries that were individually sequenced. MinION sequencing generated approximately 1.4, 0.16, and 0.15 million reads for ASFV, CSFV, and FMDV libraries, respectively. The amplification and sequencing procedures, performed at the SCVL in Mongolia, were rapid and took as little as 1 h (CSFV and FMDV) and up to 6h (ASFV). Approximately 100%, 93%, and 91% of the total reads from each library were successfully mapped to their respective ASFV, CSFV, and FMDV genomic regions (Figure 2a–c). A small proportion of reads from the CSFV and FMDV libraries (1% and 9%, respectively) matched those of other organisms, likely due to co-infections or environmental contamination at the time of sample collection and/or processing.

A more detailed analysis of the sequenced fragments revealed that almost 100% of ASFV- and FMDV-matching reads were assigned to a unique pathogen strain (Figure 2a,c). On the other hand, CSFV-matching reads were assigned primarily to CSFV Alfort/187, CSFV, and Pestivirus C (Figure 2b). About 6% of the reads from the CSFV library were assigned to other pestiviruses (Figure 2b). Considering the much lower taxonomic placement score of these reads compared to those classified as CSFV or Pestivirus C (Table 3), these reads either represent false-positive taxonomic assignments or infection of swine with other pestiviruses such as bovine viral diarrhea virus [34,35]. Further analysis of the sequencing data with the CLC Genomics Workbench, where reads were mapped to their respective reference genomes, validated the initial genomic identification of the three transboundary pathogens. Using this pipeline, we obtained a 262 bp sequence with 1.3 × 10^6^-fold coverage for ASFV, a 291 bp sequence with 1.11 × 10^5^-fold coverage for CSFV, and a 330 bp sequence with 1.10 × 10^5^-fold coverage for FMDV. We were, however, unable to differentiate between the three potential CSFV strains and other pestiviruses that were identified through the MinION analysis workflow as described above. Nonetheless, either approach successfully identified the targeted pathogens, demonstrating the suitability of this workflow for rapid and detailed identification of ASFV, CSFV, and FMDV.

**Figure 3:**
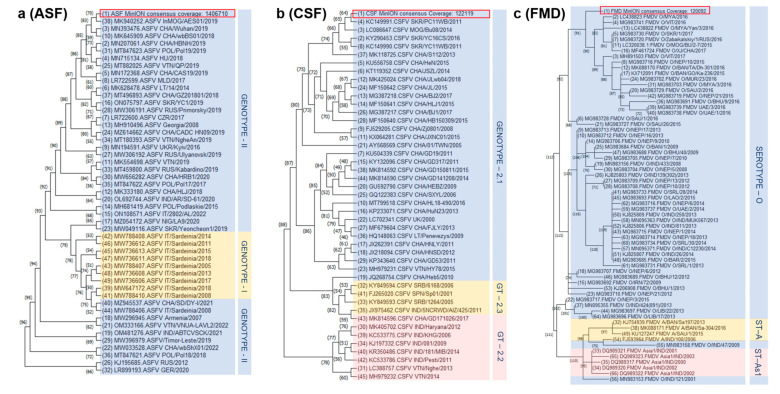
Phylogenetic trees of ASFV (**a**), CSFV (**b**), and FMDV (**c**) field isolates based on partial p72 (**a**), domain II of IRES (internal ribosomal entry site; (**b**)) and 5′ UTR (**c**), respectively. The Molecular Evolutionary Genetics Analysis (MEGA) Version 11.0.13 was used to create the phylogenetic trees. Genotype (GT) and serotype (ST) clusters are differentiated in each panel by color: (**a**) ASFV GT-I (yellow) and GT-II (blue); (**b**) CSF GT-2.1 (blue), GT-2.2 (pink) and GT-2.3 (yellow); (**c**) FMD ST-O (blue), ST-A (yellow) and ST-Asia-1 (As1; (pink).

TADs including ASF, CSF, and FMD have been frequently reported in Mongolia in 2019, 2007–2015, and 2000–2022, respectively. The most predominant TAD in Mongolia is FMD, with serotypes O, A, and Asia-1 being present in livestock including camels [3]. ASFV and CSFV were also identified and were caused by genotype II and sub-genotype 2.1b, respectively [4,5]. In order to determine the genetic proximity of the ASFV, CSFV, and FMDV isolates to those that were previously identified elsewhere, we conducted maximum likelihood (ML) phylogenetic analyses using the short genomic sequences obtained from these pathogens (Figure 3). The phylogenetic trees revealed that ASFV, CSFV, and FMDV are closely related with other viruses of the same genotype/serotype circulating in the same geographic area where they were isolated (Figure 3). The ASFV sequence (Mongolia, 2019) is part of a major genotype II cluster that is populated mainly by isolates from Eastern Asia, Eastern Europe, and Russia (Figure 3a). The CSFV isolate (Mongolia, 2015) is part of a genotype 2.1 cluster mainly encompassing Eastern Asian CSFV isolates, and more closely associated to those that were identified in South Korea (2011), Mongolia (2014), Sri Lanka (2011, 2016), and China (2013) (Figure 3b). The FMDV sequence (Mongolia, 2018) is part of a phylogenetically related serotype O cluster represented mainly by Southern and Southeastern Asian, and Middle Eastern FMDV isolates (Figure 3c).

## 4. Discussion

Next-generation sequencing (NGS) has become a valuable tool for the diagnosis of TADs and for the spatiotemporal tracking of pathogens [33]; (RT-) PCR has been most widely used for routine diagnosis in veterinary laboratories. The advantage of using NGS together with (RT-) PCR for TAD diagnostics is that sequencing information may help to promptly identify detailed pathogen features that are not provided by other diagnostic tools. Hence, this work was performed as a proof-of-concept study to investigate the suitability of portable MinION sequencing for point-of-care (POC) diagnosis of ASF, CSF, and FMD in Mongolia, a low-resource country.

Using (RT-) PCR plus NGS on a portable sequencer such as the MinION for the rapid identification of ASFV, CSFV, and FMDV isolates from field samples can help improve POC diagnostics for TADs and guide authorities in the implementation of mitigation strategies. Currently available POC diagnostics typically rely on immunoassays for antigen and antibody detection, which present a number of limitations including cross reactivity, lower sensitivity and specificity [36]. Sequencing-based POC diagnostics have the advantage of generating valuable information such as the rapid determination of pathogen classification and the identification of genotype/serotype/strain in a short period of time.

**Figure 4:**
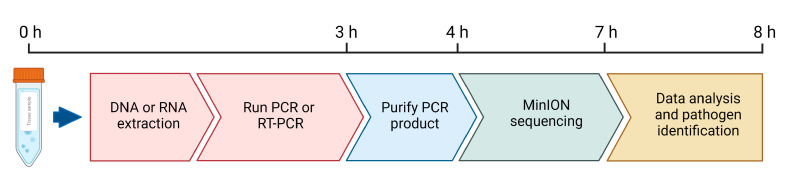
Timeline for pathogen identification. Shown are the steps from tissue sample collection and preparation to sequence data analysis. The tools in biorender.com were used to make the figure.

Although only a limited number of samples were analyzed as a proof-of-concept, our results demonstrate the suitability of short amplicon sequencing targeting critical genes of important transboundary pathogens on the MinION in a low-resource country as a potential POC diagnostic tool. This methodology is capable of providing pathogen genotype/serotype/strain identification in a fast turnaround time of approximately 8 h (Figure 4). Although diagnostic tests based on (RT-) PCR are faster, the data that were obtained from MinION sequencing can be used for assessing phylogeny and for improved diagnostic confidence, which is critical for further decision-making, for example executing rapid implementation of mitigation strategies during TAD outbreaks. The MinION in the current scenario provides almost real-time detection of specific viruses and their genotypes/serotypes and can be applied in the field. It is quite possible to further optimize the bioinformatic tools for real-time phylogenetic analysis in a field setting in the near future.

The limit of short amplicon sequencing is that it does not allow thorough molecular characterization of a particular pathogen compared to whole genome sequencing protocols that could also be employed on the MinION. However, the advantage here is that the short amplicon sequencing provides relatively rapid and reliable information when targeting critical genes/regions of pathogens that (RT-) PCR does not provide. Although the cost of this methodology compared to other POC diagnostic tests is higher, in the face of a TAD outbreak, the cost of the test becomes less of an issue since rapid (within 8 h) and accurate testing are the most critical parameters.

Thus, our results show that sequencing short fragments of conventional (RT-) PCR products is a reliable approach for rapid POC diagnostics for ASFV, CSFV, and FMDV in low-resource countries.

## Figures and Tables

**Table 1:** Sample information.

Pathogen	Virus Geno/Serotype	Tissue	Collection Date	Sample Origin	Animal Species
ASFV	Genotype II	Spleen	January 2019	Bulgan province	Domestic pig
CSFV	Genotype 2.1b	Blood	April 2015	Khan-Uul District Ulaanbaatar	Domestic pig
FMDV	Serotype O	Tongue epithelium	January 2019	Dornod province	Domestic Cattle

**Table 2:** **The** World Organization of Animal Health (WOAH)-recommended (RT-) PCR primers for the amplification of target genes of ASFV, CSFV, and FMDV.

Pathogen	Position	Forward Primer	Reverse Primer	Length
ASFV	p72	5′-AGTTATGGGAAACCCGACCC-3′	5′-CCCTGAATCGGAGCATCCT-3′	257 bp
CSFV	IRES	5’-ATGCCCACAGTAGGACTAGCA-3′	5’-TCAACTCCATGTGCCATGTAC-3’	284 bp
FMDV	5′ UTR	5′-GCCTGGTCTTTCCAGGTCT-3′	5′-CCAGTCCCCTTCTCAGATC-3′	328 bp

**Table 3:** The number of mapped reads with the CSFV sequence analysis.

Row Labels	Count of Reads	Average of Score
Pestivirus C	42,309	1010.36
Classical swine fever virus—Alfort/187	36,259	997.24
Classical swine fever virus	18,808	1087.15
Pestivirus	4612	312.34
Aydin-like pestivirus	518	241.08
Bovine viral diarrhea virus 1	242	430.90
Pestivirus giraffe-1 H138	77	336.71
Border disease virus	20	392.25
Bovine viral diarrhea virus 2	7	165.57
Bovine viral diarrhea virus 3 Th/04_KhonKaen	7	179.29
Pronghorn antelope pestivirus	1	169.00

## Data Availability

Data available upon request.
